# Congenital Superior Sternal Cleft Repair Using Primary Closure

**DOI:** 10.4274/balkanmedj.galenos.2018.2018.0955

**Published:** 2019-02-28

**Authors:** Yekta Altemur Karamustafaoğlu, Fazlı Yanık, Yener Yörük, Ümit Nusret Başaran

**Affiliations:** 1 Department of Thoracic Surgery, Trakya University School of Medicine, Edirne, Turkey; 2 Department of Pediatric Surgery, Trakya University School of Medicine, Edirne, Turkey

Sternal cleft is a rare congenital chest malformation with an incidence of <0.15% and is predominant in girls (1). It is most commonly diagnosed in the neonatal period and often associated with other congenital anomalies. However, an isolated sternal cleft with no associated abnormality is uncommon. Sternal cleft can be partial or complete, and the partial defect leads the superior or the inferior sternum part.

A full-term newborn aged 42 days and weighing 3370 g with a superior sternal cleft was transferred to our clinic. There was no antenatal diagnosis. The patient was initially followed up in the pediatric intensive care unit due to respiratory distress without intubation. Computed tomography and three-dimensional reconstruction revealed a superior U-shaped sternal cleft with displacement of the medial ends of the clavicles (Figure 1a, 1b). Laboratory test results were normal, and the baby was in good condition. There was paradoxical movement of the 4×4 cm defect without any other associated anomaly on examination and ultrasonography (Figure 1c). Heartbeats were easily visible through the normal skin over the cleft area. During the operation, the skin flaps separated from the pericardium and the pectoralis major muscles were lateralized from their adhering medial chondral insertion (Figure 2a). The “U-shaped” fused portion of the sternal edges and the xiphoid were resected vertically to the incision line on each side until the entire surface of the edges could be fitted throughout the entire length. Then, the posterior perichondrium of the sternum was approached with 2/0 Vicryl sutures from the lower to the upper end (Figure 2b). The heart rate, oxygen saturation, and blood pressure were monitored for 3 min, and then the sutures were tied. A few cardiac arhythm  were observed. These were well tolerated and after disappeared. Then, the sutures were tight and the pectoral muscles were fused on the midline. Postoperative period was uneventful. The patient was discharged 8 days later. Six months after the surgery, the baby is healthy and growing well, and the sternum is stable. Written informed consent was obtained from the parents.

Sternal cleft is a rare anomaly of the chest wall (1). According to the literature, there are reports indicating that it affects people of older ages, adolescents, and in puberty, and different methods have been used for correction, including otologic graft or material. However, an isolated sternal cleft with no associated abnormality is sporadic (2). Early surgical management of correcting the sternal cleft is proposed in the neonatal period, allowing for primary closure, preferably before 3 months of age (3). For the extremely rare form of the isolated superior sternal cleft reported here, closure of the defect using primary suture was performed in the newborn.

## Figures and Tables

**Figure 1:**
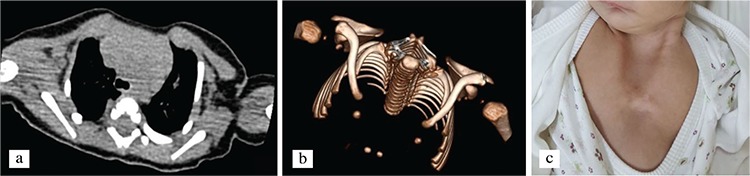
Axial section of preoperative computed tomography scan showing the partial superior sternal cleft with the complete lack of fusion of the sternal bars (a). Three-dimensional reconstruction showing the split sternum bones with the ossification center (b). Preoperative view of the anterior chest Wall (c).

**Figure 2:**
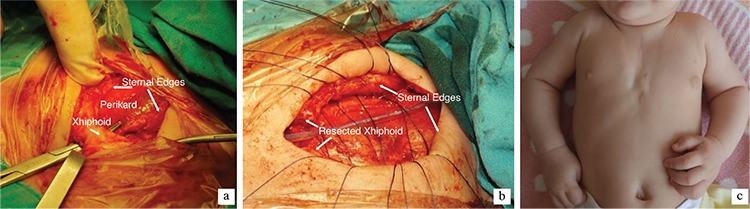
Intraoperative view revealing the U-shaped sternal defect (a). Approximation of the sternal bars (b). Postoperative appearance of the chest wall after 6 months (c).
